# IL-1 Contributes to the Anti-Cancer Efficacy of Ingenol Mebutate

**DOI:** 10.1371/journal.pone.0153975

**Published:** 2016-04-21

**Authors:** Thuy T. Le, Kresten Skak, Kate Schroder, Wayne A. Schroder, Glen M. Boyle, Carly J. Pierce, Andreas Suhrbier

**Affiliations:** 1 Inflammation Biology, and Cancer Drug Mechanism Laboratories, QIMR Berghofer Medical Research Institute, Brisbane, Queensland, Australia; 2 Leo Pharma, Copenhagen, Denmark; 3 Inflammasome Laboratory, Institute for Molecular Bioscience and Australian Infectious Diseases Research Centre, University of Queensland, Brisbane, Queensland, Australia; Columbia University, UNITED STATES

## Abstract

Ingenol mebutate is approved for the topical treatment of actinic keratoses and may ultimately also find utility in treating skin cancers. Here we show that relapse rates of subcutaneous B16 melanoma tumours treated topically with ingenol mebutate were not significantly different in C57BL/6 and Rag1^-/-^ mice, suggesting B and T cells do not play a major role in the anti-cancer efficacy of ingenol mebutate. Relapse rates were, however, significantly increased in MyD88^-/-^ mice and in C57BL/6 mice treated with the anti-IL-1 agent, anakinra. Ingenol mebutate treatment induces a pronounced infiltration of neutrophils, which have been shown to have anti-cancer activity in mice. Herein we provide evidence that IL-1 promotes neutrophil recruitment to the tumour, decreases apoptosis of infiltrating neutrophils and increases neutrophil tumour killing activity. These studies suggest IL-1, via its action on neutrophils, promotes the anti-cancer efficacy of ingenol mebutate, with ingenol mebutate treatment causing both IL-1β induction and IL-1α released from keratinocytes.

## Introduction

Ingenol mebutate (Picato^®^) is an approved topical drug for field treatment of actinic keratoses (AKs) [1,2,3]. AKs are lesions usually found on sun-exposed skin caused by UV exposure and are characterised by atypically proliferating keratinocytes [4]. In about 8% of cases, AKs can progress to invasive squamous cell carcinomas (SCCs) [5], the second most common form of skin cancer. Removal of AKs and mutated keratinocytes by ingenol mebutate treatment thus seeks to reduce the subsequent risk of developing SCCs [1,6,7,8]. Ingenol mebutate may ultimately also find utility in treating other skin cancers [9,10], with efficacy shown in human studies for SCCs and basal cell carcinomas [11,12,13]. Ingenol mebutate is derived from the sap of *Euphorbia peplus* and activates protein kinase C (PKC) [14,15]. *In vivo* topical drug application induces primary necrosis of (i) keratinocytes, including patches of keratinocytes bearing p53 mutations and (ii) subcutaneous tumours cells growing beneath the treatment site [16,17]. Topical treatment in humans [7,18,19] and mice [16] results in a rapid onset transient erythema and a pronounced recruitment of neutrophils [17,20,21]. Neutrophils recruited and stimulated by ingenol mebutate also appear to mediate anti-cancer activity by significantly reducing tumour-relapse rates in mouse tumour models, including the B16 melanoma model [21].

To investigate the role of adaptive and innate immune responses to the anti-cancer efficacy of ingenol mebutate, we investigated relapse rates after topical treatment of subcutaneous B16 tumours with ingenol mebutate in a series of genetically modified mice. Although T and B cell responses did not appear to play a major role, we provide evidence that IL-1 plays a significant role in the anti-cancer efficacy of ingenol mebutate.

## Materials and Methods

### Animal ethics statement

All mouse work was conducted in accordance with good animal practice as defined by the National Health and Medical Research Council of Australia. The mouse work was approved by the QIMR Berghofer Medical Research Institute animal ethics committee. Mice were euthanized when tumours reached 100 mm^2^, a tumour size that does not cause excessive distress to the animals.

### Mice, tumour inoculation and ingenol mebutate treatment

Female mice, 8–12 weeks old were injected with B16 melanoma cells (4–5x10^5^ cells/mouse in 50 μl medium) by shallow s.c. injection. B16 cells were obtained from the ATCC (CRL-6322) passaged no more than 10 times and shown to be Mycoplasma negative by MycoAlert^™^ Mycoplasma Detection Kit (Lonza, Basel, Switzerland) and Hoechst staining [22]. When the mean tumour size reached 10–20 mm^2^ (2–4 days post injection) mice were chosen so that each group had a similar mean tumour size and tumour size distribution; mice with tumour sizes that could not be appropriately matched were removed. The tumour sites were then treated topically once with 10 μl placebo or 0.1% ingenol mebutate gel daily for 2 consecutive days (treatment initiation deemed to be day 0). When tumours reached 100 mm^2^ mice were euthanized using carbon dioxide gas. The 0.1% gel comprises 1 mg/g of ingenol-3 angelate in an excipient containing isopropyl alcohol, hydroxyethylcellulose, citric acid monohydrate, sodium citrate, benzyl alcohol and purified water. This dose and schedule was chosen as previous work had shown it provided a 50–100% regression rate in the B16 mouse model [16,21].

C57BL/6 mice were purchased from ARC, Perth, Australia. MyD88^-/-^ (B6.129P2(SJL)-*Myd88*^*tm1*.*1Defr*^/J), Rag1^-/-^ (B6.129S7-*Rag1*^*tm1Mom*^/J) and μMT (B6.129S2-*Igh*-6^*tm1Cgn*^/J) mice on a C57BL/6 background were obtained from Jackson Laboratory (Bar Harbor, ME, USA), and were bred in-house at QIMR B. FcR-common gamma chain-deficient mice (FcγR^-/-^) (B6.129P2-*Fcer1g*^*tm1Rav*^ N12) on a C57/BL6 background were purchased from Taconic (Hudson, NY, USA).

### Anakinra treatment

Kineret (anakinra) SOBI (Swedish Orphan Biovitrum) was given i.p. at 1 mg /mouse (in 100 μl in PBS) daily, day 0 (3–4 hours before ingenol mebutate treatment initiation) through to day 7. The same volume of PBS was given to control mice.

### Histology and immunohistochemistry

Hematoxylin and eosin (H&E), ApoTag staining (ApoTag Plus Peroxidase *In situ* Apoptosis Detection Kit, Millipore) and anti-Ly6G staining (using rat anti-mouse Ly6G; catalogue number NMP-R14; Abcam, Cambridge, MA, USA) were undertaken as described previously [23,24]. Slides were digitally scanned by using a Scan ScopeXTdigital slide scanner (Aperio, Vista, CA) and image analyses of sections in duplicate were undertaken using Aperio ImageScope software (v10) and the Positive Pixel Count v9 algorithm using default settings.

### Killing assays

B16 cells (5x10^3^) were seeded into 96 well flat plates in 100 μl DMEM supplemented with 5% fetal calf serum and 10 mM HEPES pH 7.2. The next day (day 1) bone marrow cells were collected, washed once, and added within 45 mins of collection at the indicated effector to target ratios with 6 replicates. Anakinra (100 μg/ml final) or IL-1β (Shenandoah Biotechnology Inc., Warwick, PA, USA) (40 ng/ml final) was then added, followed by ingenol mebutate (40 ng/ml final). The next day (day 2) suspension cells were removed by inverting the plates onto tissue paper and fresh media was added. After a further 2 days incubation (day 4), medium was removed by inverting the plates onto tissue paper and the cell were fixed and stained with crystal violet. The plates were dried, 100 μl methanol added per well and absorbance measured at 595 nm. Killing was calculated relative to B16 cells without effectors after subtraction of background.

### IL-1α and IL-1β protein measurements

Mice with B16 tumours were treated with ingenol mebutate day 0 and 1, treatment sites were excised at the indicated times, homogenized (using Precellys24 tissue homogenizer and ceramic beads) in PBS supplemented with 0.1% Igepal CA-630 nonionic detergent (Sigma) and protease inhibitor cocktail (Roche). IL-1α and IL-1β levels were measured in the supernatants using BD™ Cytometric Bead Array Flex Set reagents (BD Biosciences).

### Keratinocyte cultures and anti-IL-1α immunoblotting

Human adult epidermal keratinocytes (HEKa-APF, Cascade Biologics, OR, USA) were cultured as per supplier’s recommendations on coating matrix and in low calcium medium (EpiLife with S7) without serum (Gibco). Cells we grown to confluence in 6 well plates, calcium was then added to a final concentration of 1 mM and the cells cultured for 48 hours. Cells were then treated with the indicated concentrations of ingenol mebutate for 16 hours in 1 ml of EpiLife medium containing 1 mM calcium. The supernatants were harvested, spun at 1000 g for 5 mins, and proteins in the supernatant precipitated using methanol as described [25]. Precipitates were then analysed by Western blotting using an anti-IL-1α antibody (Abcam; catalogue number 9614) and goat anti-rabbit HRP secondary antibody (Merck Millipore). Blots were scanned using ImageQuant LAS 500.

### Statistics

Statistical analysis was performed using IBM SPSS Statistics (version19). Survival and relapse rates are represented as Kaplan-Meier plots, with statistical significance determined by log-rank (Mantel-Cox) tests. For other data sets the t test was used if the difference in the variances was <4, skewness was >-2, and kurtosis was <2; where the data was nonparametric and difference in variances was <4, the Mann Whitney U test was used, if >4 the Kolmogorov-Smirnov test was used. A 2 way ANOVA was used for ApoTag staining, with data showing a parametric distribution and differences in variance <4.

## Results

### Relapse rates in mice deficient in T and B cell responses

Both anti-cancer T cells and anti-cancer antibodies are induced after topical ingenol mebutate treatment (and regression) of a number of subcutaneously grown tumours in mice [9,10,21]. Experiments using the LK2 squamous cell carcinoma line grown in *Foxn1*^*nu*^ mice also suggesting a role for antibodies and antibody dependent cellular cytotoxicity (ADCC) in preventing relapse [21]. To investigate further the contribution of adaptive immune responses to the anti-cancer activity of ingenol mebutate, B16 tumours were grown subcutaneously to 10–20 mm^2^ in Rag1^-/-^ mice (C57BL/6 background), which are unable to generate adaptive B or T cell responses, and in control C57BL/6 mice. The tumour sites were then treated twice topically (day 0 and day 1) with ingenol mebutate or placebo gel, and tumour growth was monitored over time. Relapse rates were not significantly different between Rag1^-/-^ and C57BL/6 mice (Figure A in S1 File), suggesting neither B nor T cells play a major role in the anti-tumour efficiency of ingenol mebutate in the B16 model.

Further experiments to investigate the role of (i) ADCC [21] using Fc receptor common gamma chain deficient mice (FcγR^-/-^) mice (Figure B in S1 File) and (ii) antibodies using B cell deficient μMT mice (Figure C in S1 File), were inconclusive as growth rates of B16 (in the absence of ingenol mebutate treatment) were different in these mice.

### Increased relapse rates in MyD88^-/-^ mice

To investigate further the contribution of innate immune responses [21] to the anti-cancer efficacy of ingenol mebutate treatment, B16 cells were grown subcutaneously to 10–20 mm^2^ in MyD88^-/-^ and C57BL/6 mice and were then treated twice (day 0 and day 1) with ingenol mebutate or placebo gel, and tumour growth monitored over time.

**Fig 1:**
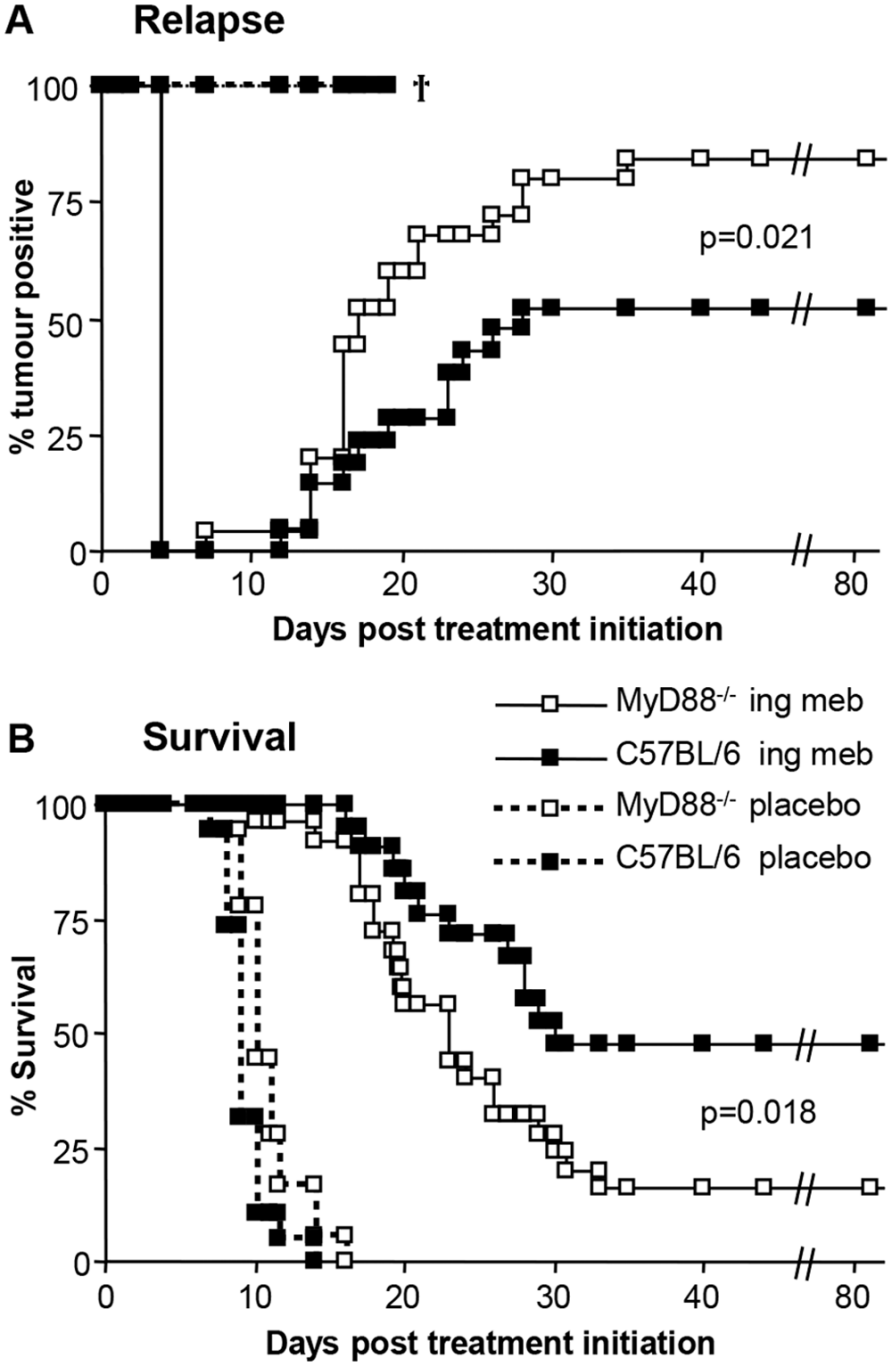
Relapse and survival following ingenol mebutate treatment of B16 tumours grown in MyD88^-/-^ and C57BL/6 mice. (A) Relapse rates following (i) ingenol mebutate treatment of B16 tumours grown in MyD88^-/-^ mice (n = 25), (ii) ingenol mebutate treatment of B16 tumours grown in C57BL/6 mice (n = 21), (iii) placebo treatment of B16 tumours grown in MyD88^-/-^ mice (n = 18) and (iv) placebo treatment of B16 tumours grown in C57BL/6 mice (n = 19). Mice were scored positive when a tumour was clearly visible (≥1–2 mm in diameter). Data from two independent experiments. Ingenol mebutate treatment groups were significantly different p = 0.021, log-rank (Mantel-Cox) test. (B) Survival rates of the same mice described in A; mice were euthanized when tumours reached 100 mm^2^. Ingenol mebutate treatment groups were significantly different p = 0.018, log-rank (Mantel-Cox) test.

The relapse rates in MyD88^-/-^ mice were significantly higher (84%) than in C57BL/6 mice (50%) post ingenol mebutate treatment (Fig 1A), with an ensuing significant reduction in survival from 50% in C57BL/6 to 16% in MyD88^-/-^ mice (Fig 1B).

In contrast to a previous report [26], we observed no significant differences in the growth of B16 in MyD88^-/-^ and C57BL/6 mice in placebo treated mice (Figure D in S1 File). Survival rates were actually marginally (but not significantly) increased in MyD88^-/-^ mice when compared with C57BL/6 in the absence of ingenol mebutate (Fig 1B, compare placebo groups). The latter two observations indicate that the increased relapse rates in MyD88^-/-^ mice post ingenol mebutate treatment were not due to inherently more robust growth of B16 tumours in MyD88^-/-^ mice.

### Anakinra treatment increased relapse

**Fig 2:**
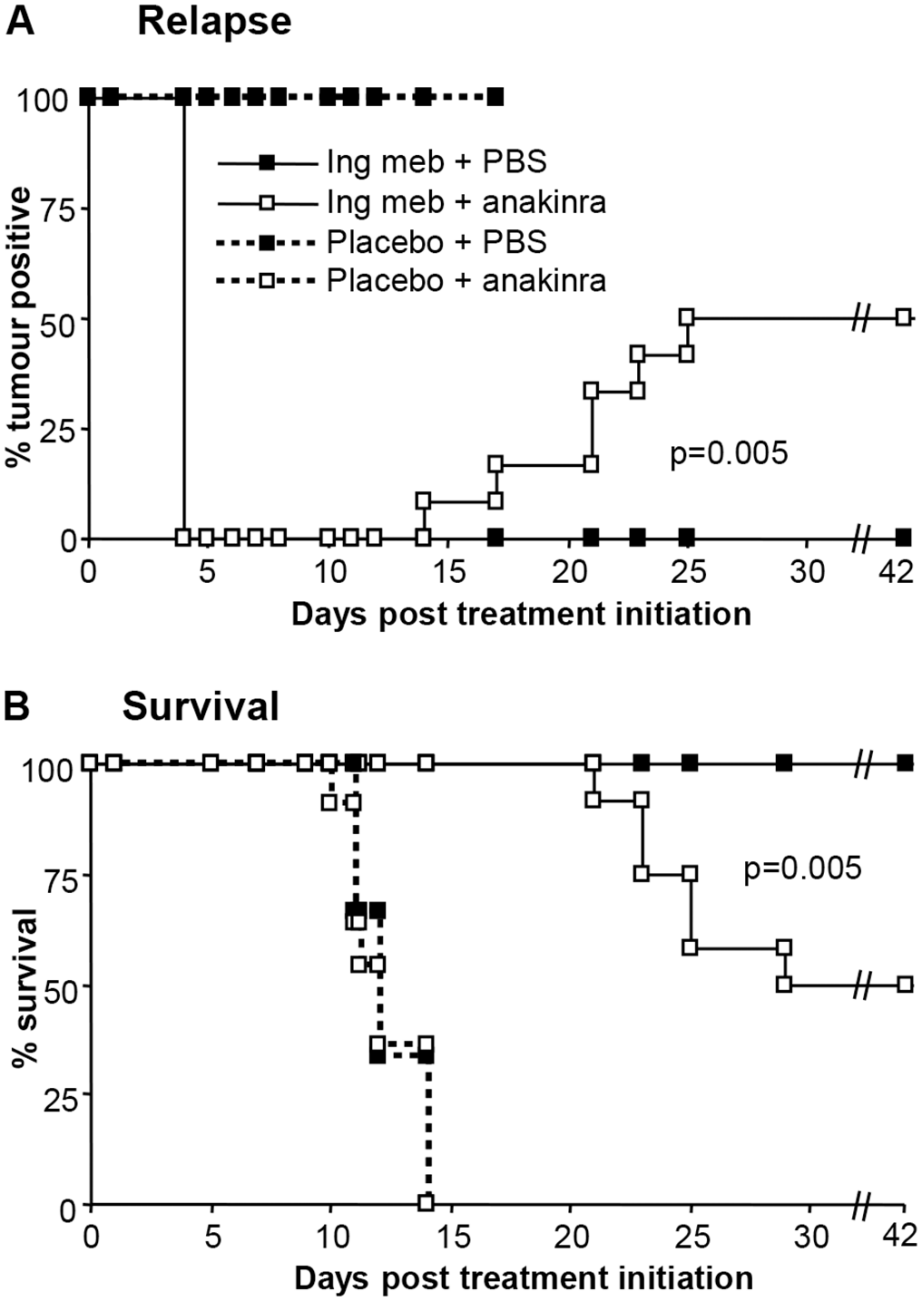
Relapse and survival following ingenol mebutate treatment of B16 tumours grown in C57BL/6 mice treated with anakinra. (A) Relapse rates after C57BL/6 mice bearing B16 tumours were treated with ingenol mebuate or placebo, and received daily injections of PBS or anakinra, days 1–7. (n = 9–12 mice per group). Mice were scored positive when a tumour was clearly visible (≥1–2 mm in diameter). Statistics compared + anakinra with + PBS in ingenol mebutate treated groups using the log-rank (Mantel-Cox) test. (B) Survival of the mice described in A; mice were euthanized when tumours reached 100 mm^2^. Statistics as in A.

MyD88 is involved in signal transduction for both Toll-like receptors and the IL-1 receptor, and IL-1β mRNA is induced in mice after topical ingenol mebutate treatment [21]. Keratinocytes express high levels of constitutive IL-1α, which is released after topical application of phorbol ester [27]; phorbol ester, like ingenol mebutate, activates PKC [14]. The use of IL-1 deficient mice is complicated by the very different growth rates of B16 in these animals when compared with wild-type mice [28]. Thus to investigate the role of IL-1, mice were treated with anakinra, a recombinant IL-1 receptor antagonist used in the treatment of rheumatoid arthritis. Anakinra treatment significantly increased relapse rates from 0% to 50% (Fig 2A) and decreased survival from 100% to 0% (Fig 2B), suggesting that IL-1 plays a role in the anti-tumour efficacy of ingenol mebutate. Anakinra treatment did not affect the growth of established B16 tumours *in vivo* (Figure E in S1 File), consistent with previous findings [29,30,31].

### Anakinra and neutrophil recruitment and apoptosis

**Fig 3:**
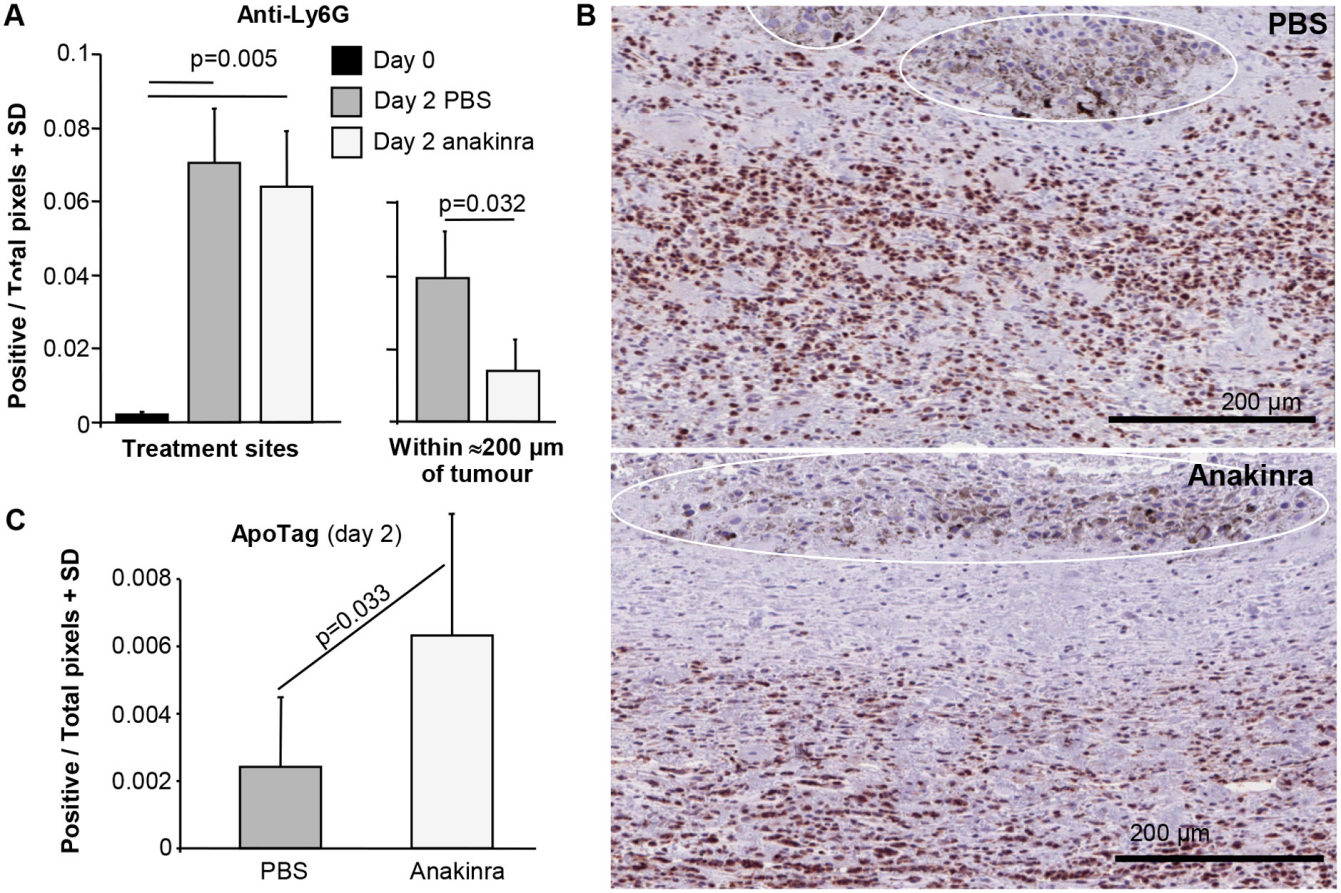
Neutrophil recruitment and apoptosis. (A) Neutrophil recruitment to treatments sites (left bar chart) and to within ≈200 μm of tumour (right bar chart). Treatment sites; day 2 post initiation of ingenol mebutate treatment, treatment sites were excised and processed for immunohistochemistry and stained with the neutrophil marker, anti-Ly6G. Slides were scanned and analysed by Aperio Pixel count software for brown staining (default settings) excluding areas containing tumour (as melanosomes provide a false positive signal). Two sections per mouse, 6 mice per group. Statistics by Kolmogorov-Smirnov tests (differences in variance between groups was >4). Within ≈200 μm of tumour; in sections where the tumour mass could be readily identified (by the presence of black melanosomes), brown staining surrounding the tumour (within ≈200 μm) was quantitated as above. One section per mouse, 4–5 mice per group. Statistics by Mann Whitney U test (non-parametric data distribution and differences in variance <4). (B) Images illustrating the reduced density of anti-Ly6G staining neutrophils (brown stain) within ≈200 μm of the tumour mass in anakinra versus PBS treated mice. The tumours are delineated by white lines and identified by the presence of black melanosomes. Sections are oriented with the skin (not shown) at the top, with the tumours located in the dermis. (C) ApoTag staining of the sections described in A. Six mice per group, 2/3 sections per mouse, statistics by 2 way ANOVA (parametric data distribution and differences in variance <4, drug and mouse as fixed factors, 2/3 sections per mouse as dependent variables). (Examples of the staining are shown in Figure F in S1 File).

Topical ingenol mebutate treatment results in a pronounced recruitment of neutrophils to the treatment site [9,17,20,21], which herein was quantitated using anti-Ly6G antibody staining, immunohistochemistry and image analysis (Aperio Positive Pixel count). As expected, significant recruitment of neutrophils to the treatment site (peaking on day 2 post treatment initiation) was observed (Fig 3A, Treatment site, compare day 0 with day 2 PBS). Although IL-1 has been shown to promote neutrophil recruitment in several settings [32,33,34] anakinra treatment did not significantly affect recruitment to the treatment site (Fig 3A, Treatment site, compare day 2, PBS versus anakinra). However, in sections where the tumour could be clearly seen, the density of neutrophils within ≈200 μm of the tumour mass was marginally (>2 fold), but significantly, lower in anakinra treated animals (Fig 3A, Within ≈200 μm of tumour, compare day 2, PBS versus anakinra). Examples of the latter are shown in Fig 3B, with neutrophils staining brown and the tumour mass readily identifiable by the black melanosomes (Fig 3B, white ovals). Anakinra thus appeared to reduce neutrophil recruitment to the tumour, with tumour-derived IL-1β previously reported to recruit anti-cancer neutrophils to the tumour [35].

IL-1 has been reported to inhibit neutrophil apoptosis [36,37,38]. Parallel sections to those described above were therefore stained with ApoTag and image analysis (as above) of neutrophil rich areas undertaken to measure the extent of apoptosis. Examples of the Apotag staining are shown in Figure F in S1 File. Anakinra treatment more than doubled the ApoTag staining density in neutrophil rich areas in treatment sites in the dermis 2 days post ingenol mebutate treatment initiation (Fig 3C), suggesting IL-1 promotes the survival of neutrophils recruited by ingenol mebutate treatment.

### Ingenol mebutate and IL-1β promote killing of B16 cells *in vitro*

**Fig 4:**
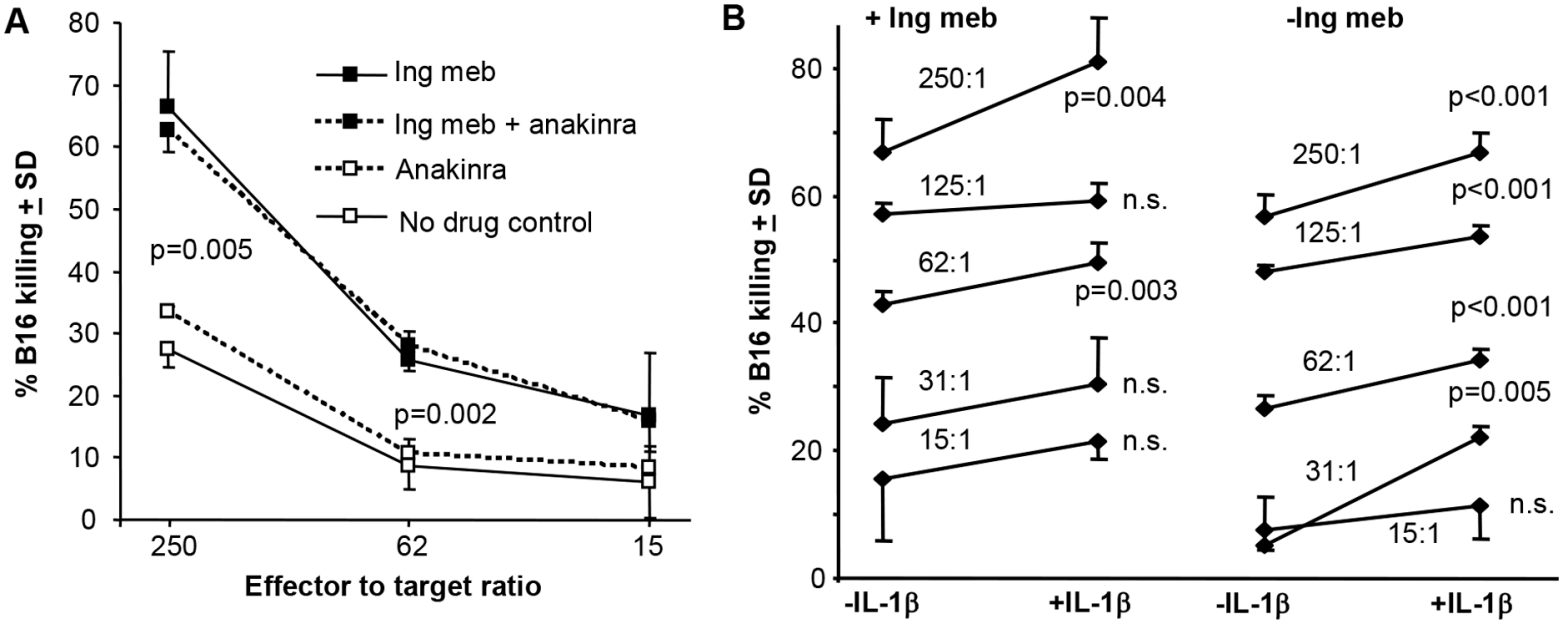
*In vitro* killing of B16 tumour cells in the presence of ingenol mebutate, anakinra and/or IL-1β. (A) Bone marrow cells (≈40% neutrophils) were incubated with B16 cells in 96 well plates (6 replicates) at the indicated effector to target ratios and cell killing calculated relative to B16 cells with no drug or effectors. Statistics by Kolmogorov-Smirnov (difference in variance >4) p = 0.005, and Mann Whitney U test (non-parametric distribution and difference in variance <4) p = 0.002. Ingenol mebutate (40 ng/ml final) and/or anakinra (100 μg/ml final) was present during the assay. (B) As for A except a large range of effector to target ratios (i.e. 15:1 to 250:1) were used and IL-1β (40 ng/ml final) was added. Statistics by Kolmogorov-Smirnov (for data sets where difference in variance was >4) and t tests (for data sets with parametric distribution and differences in variance <4).

We have previously shown that human neutrophils show enhanced killing of human melanoma cells *in vitro* in the presence of ingenol mebutate [21]. To investigate further the ability of ingenol mebutate to promote tumour killing by neutrophils, we developed a murine system using B16 cells and bone marrow cells, which comprise ≈40% neutrophils, ≈40% B cells, ≈10% monocytic cells and a small number of other cell types. Ingenol mebutate was consistently able significantly to promote killing of B16 cells by these cells by ≥ 2 fold (Fig 4A, p = 0.005 and 0.002). This killing was not inhibited in the presence of anakinra (Fig 4A, dashed lines) suggesting that neither ingenol mebutate-stimulated neutrophils [39] nor B16 cells generate sufficient IL-1 to stimulate tumour cell killing [35]. However, in the presence of exogenously added IL-1β, killing was marginally but consistently, and often significantly, increased in both the presence and absence of ingenol mebutate (Fig 4B). These data support the view that IL-1 improves the anti-cancer activity of neutrophils *in vitro*, consistent with previous reports showing that IL-1 can promote degranulation and respiratory burst activity in human neutrophils [40,41]. (We were unable formally to exclude the possibility that other cells in bone marrow were responsible for the anti-cancer cell activity, as purification of murine neutrophils resulted in inconsistent results).

### IL-1 protein levels at the ingenol mebutate treatment sites

**Fig 5:**
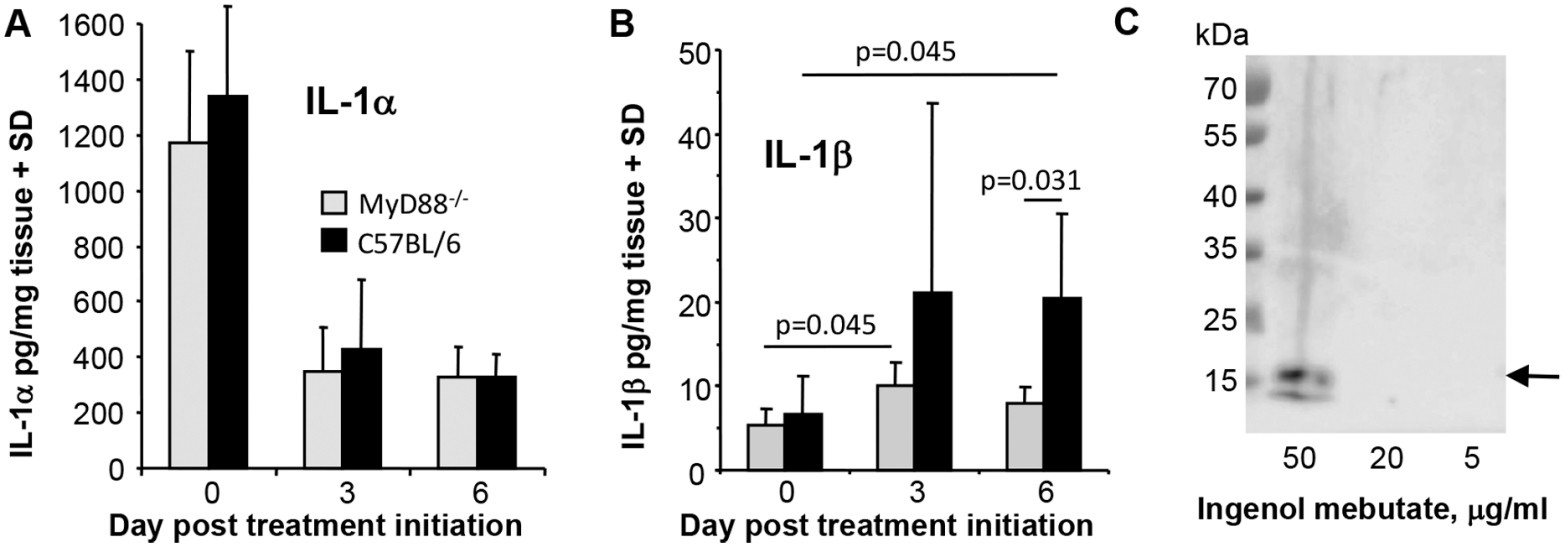
IL-1α and IL-1β protein levels after ingenol mebutate treatment. (A, B) C57BL/6 and MyD88^-/-^ mice with B16 tumours were treated topically with ingenol mebutate day 0 and 1, treatment sites were excised and IL-1α and IL-1β levels were measured in extracts using BD BD™ Cytometric Bead Array (n = 6 mice per group and time point). Statistics by Kolmogorov-Smirnov tests (differences in variance >4). (C) Cultured adult human keratinocytes were treated with the indicated concentration of ingenol mebutate for 16 hours and the supernatants analysed by Western using an anti-IL-1α antibody. Arrow indicates the position of the 18 kDa bioactive form of IL-1α.

The data so far supports the view that IL-1 promotes the anti-cancer efficacy of ingenol mebutate by promoting neutrophil anti-cancer activity, with MyD88 required for IL-1 receptor signal transduction [42]. To gain further insights into IL-1 production, tissue IL-1α and IL-1β protein levels at the treatment sites were quantitated before and after ingenol mebutate treatment. IL-1α protein levels at the treatment sites were high prior to treatment (Fig 5A), with IL-1α expression restricted to skin rather than the B16 tumour (Figure G in S1 File). This observation is consistent with the known high constitutive pro-IL-1α protein expression in keratinocytes [27]. IL-1α protein levels dropped significantly (≈3 fold) post treatment (Fig 5A), likely associated with extensive keratinocyte necrosis induced within 6–24 h of ingenol mebutate treatment [17]. This pattern of IL-1α protein expression was similar in MyD88^-/-^ mice (Fig 5A, MyD88^-/-^; Figure G in S1 File).

IL-1β protein levels in C57BL/6 mice were low prior to ingenol mebutate treatment and increased (≈3 fold) after ingenol mebutate treatment, with this increase reaching significance on day 6 (Fig 5B, C57BL/6) consistent with previous mRNA data [21]. On day 6 IL-1β protein levels were also significantly higher in ingenol mebutate treated C57BL/6 mice when compared with ingenol mebutate treated MyD88^-/-^ mice (Fig 5B, day 6). Thus MyD88^-/-^ mice have a defect in IL-1β protein induction after ingenol mebutate treatment, consistent with the MyD88-dependent IL-1β induction seen in several settings [43,44].

The bead based IL-1 assays, used herein on tissue lysates, are unable to differentiate between inactive (pro-IL-1) and cleaved-active IL-1 species, with recent data suggesting pro-IL-1α also needs to be cleaved to the 18 kDa form be active at physiological concentrations [45]. Thus IL-1α released from keratinocytes [27] and/or IL-1β induction may provide IL-1 after ingenol mebutate treatment.

### Ingenol mebutate causes IL-1α release from keratinocytes *in vitro*

The considerably higher levels of IL-1α, when compared with IL-1β (Fig 5A vs 5B), might argue that IL-1α is a major player in this setting. The drop in total IL-1α levels after ingenol mebutate treatment (Fig 5A) (contemporaneous with keratinocyte necrosis [17]) suggests keratinocyte IL-1α may be released, given that other topically applied PKC activators cause IL-1α release from keratinocytes [27]. Keratinocytes constitutively express high levels of intracellular pro-IL-1α [27], which is bound to IL-1 receptor 2 (IL1R2) [46], with IL1R2 actually up-regulated after ingenol mebutate treament [15]. Production of physiologically active IL-1α is believed to require (i) release of pro-IL-1α from IL-1R2, a process mediated by caspase-1-mediated cleavage of IL-1R2, and (ii) subsequent cleavage of the released pro-IL-1α by calpain to generate physiologically bioactive 18 kDa IL-1α [45].

To determine whether ingenol mebutate treatment of keratinocytes causes the release of IL-1α, cultured primary human keratinocytes were treated with ingenol mebutate *in vitro* for 16 hours and the supernatant analysed for the presence of the 18 kDa species of IL-1α by Western. Treatment with ingenol mebutate at 50 μg/ml, but not 20 or 5 μg/ml, resulted in the release of 18 kDa IL-1α (Fig 5C, arrow). The 50 μg/ml concentration causes increases in cytosolic calcium, but is just below the concentration that causes overt cytotoxic affects in keratinocytes [47]; (we also observed no overt cell death in these cultures).

## Discussion

Herein we provide evidence that the anti-cancer efficacy of topical ingenol mebutate treatment requires both MyD88 and IL-1. Increased tumour relapse rates were seen in MyD88^-/-^ mice, and in C57BL/6 mice following anakinra (anti-IL-1) treatment, with MyD88 required for IL-1 receptor signalling [42]. Topical ingenol mebutate treatment is known to cause a pronounced recruitment of neutrophils to the treatment site [9,17,20,21], with IL-1 previously shown to promote the anti-cancer activities of neutrophils in the B16 model [35,48]. (IL-1β has no direct effect on B16 growth [35]). Neutrophils are also known to mediate anti-cancer activities in other systems [49,50]. We show herein that IL-1 affects three aspects of neutrophil behaviour following ingenol mebutate administration; (i) increased recruitment to the tumour (Fig 3B), consistent with the widely reported role of IL-1 in promoting neutrophil recruitment [32,33,34,35,51,52,53], (ii) reduced neutrophil apoptosis (Fig 3C), with IL-1 previously reported to inhibit neutrophil apoptosis [36,37,38], and (iii) increased tumour killing, consistent with the reported role of IL-1 in promoting neutrophil activation [40,41]. IL-1 and IL-1 receptor signalling thus appear to play a role in reducing relapse rates post ingenol mebutate treatment by promoting the anti-cancer activities of neutrophils.

Treatment of keratinocytes *in vitro*, with a dose of ingenol mebutate that is capable of raising intracellular calcium [47], resulted in the release of the 18 kDa bioactive form of IL-1α. Keratinocyte-derived IL-1α may thus (at least initially) be a major source of IL-1 after ingenol mebutate treatment. Lower ingenol mebutate doses did not induce IL-1α release, suggesting PKC activation (which occurs at concentrations as low as 10 ng/ml [21]) is not sufficient for IL-1α release. Increased intracellular calcium [16] might be expected to activate calpain [54], with active calpain required for cleavage of pro-IL-1α to the 18 kDa IL-1α species [45]. However, pro-IL-1α must first be released from IL-1R2, a process believed to be mediated by caspase-1-mediated cleavage of IL-1R2 [45]. Release of 18 kDa IL-1α release may suggest activation of keratinocyte caspase-1, and caspase 1 induction and activation can be detected in skin after ingenol mebutate treatment (Figure H in S1 File). Furthermore, permeabilization of the plasma membrane may be sufficient for inflammasome (and thus caspase 1) activation [55], with ingenol mebutate able to induce plasma membrane permeabilization [16,47]. However, activation of keratinocyte caspase-1 (and perhaps pyroptosis [56]) after topical ingenol mebutate treatment remains to be formally demonstrated, and is complicated by the abundance of neutrophils, which also express caspase 1.

The keratinocyte-derived IL-1α may be involved in (MyD88-dependent) induction of IL-1β (Fig 5B), with IL-1β induction by IL-1 reported previously [57,58] and tumour-derived IL-1β also shown to recruit neutrophils to the tumour [35]. As B16 cells do not make IL-1 [35], non-malignant cells in/around the tumour (e.g. stromal cells, fibroblasts and/or macrophages) may represent the source of IL-1β. After ingenol mebutate treatment *in vivo* (i) B16 tumour tissue show a ≈1.5 fold induction of IL-1β mRNA [21] and (ii) tumour treatment sites show a ≈2.5 fold increase in IL-1β protein levels (Fig 5B). Stimulated neutrophils can also make IL-1β [39,59]. Toll-like receptor engagement could also be involved in IL-1β induction [43,44], with induction of primary necrosis and haemorrhage in the tumour (evident after ingenol mebutate treatment in mice [60], see also Figure I in S1 File) potentially providing self-derived Toll-like receptor agonists [61].

Previous data showing increased relapse of LK2 tumours in SCID mice (that make little or no immunoglobulin) versus *Foxn1*^*nu*^ mice (that retain the ability to make IgM), suggested a role for anti-cancer antibodies and neutrophil ADCC in reducing relapse rates [21]. However, the data presented herein using B16 tumours grown in Rag1^-/-^ mice, suggests neither antibodies (nor T cells) play a major role in reducing relapse rates. LK2 tumours were generated in C3H/HeNCr mice (H2^k^) [62], but were grown in SCID and *Foxn1*^*nu*^ mice on a Balb/c (H2^d^) background [21]. The discrepancy between the LK2 and B16 models may thus be explained (at least in part) by allogeneic IgM responses directed against LK2 in *Foxn1*^*nu*^ mice, which would not be present in the syngeneic (and poorly immunogenic) B16 C57BL/6 model. Although IgG responses specific for the tumour are generated after ingenol mebutate treatment of B16 tumours [21] (and T7 squamous cell carcinomas [9]), the Rag1^-/-^ experiments would suggest such responses have minimal impact on relapse rates of the ingenol mebutate treated tumours. Injection of anti-B16 anti-serum (raised against B16 lysates) also did not reduce relapse rates after ingenol mebutate treatment (Figure J in S1 File), providing further support for this contention. In summary, no major role for antibodies in the anti-cancer efficacy of ingenol mebutate was apparent in the B16 C57BL/6 model.

Although many of the complex processes and interactions involved in the anti-cancer activity of ingenol mebutate remain to be established, this paper provides evidence that IL-1 (and its effect on neutrophils) is involved in the anti-cancer efficacy of ingenol mebutate. Our study also highlights the question of whether other inflammatory cytokines, for instance, TNF or IL-6 (both also induced by ingenol mebutate [21]), are also involved in the anti-cancer efficacy of ingenol mebutate.

## Supporting Information

S1 File Supporting Information. B16 relapse rates in Rag1^-/-^ mice after ingenol mebutate treatment **(Figure A).** B16 relapse, survival and growth in FcγR^-/-^ mice after ingenol mebutate **(Figure B).** B16 relapse rates in μMT^-/-^ mice after ingenol mebutate treatment **(Figure C).** Growth of B16 tumours in placebo treated MyD88^-/-^ and C57BL/6 mice **(Figure D).** Anakinra did not affect B16 growth **(Figure E).** High resolution images of ApoTag staining (**Figure F).** IL-1α levels in skin and B16 tumours **(Figure G).**) Capase 1 in skin after ingenol mebutate treatment **(Figure H).** Haemorrhage post ingenol mebutate treatment **(Figure I).** The effects of anti-B16 anti-serum on relapse rates of B16 tumours after ingenol mebutate treatment **(Figure J)**.
(PDF)
