# Supplementary material for: IL-1 Contributes to the Anti-Cancer Efficacy of Ingenol Mebutate
Source: PLoS One. 2016 Apr 21;11(4):e0153975. doi: 10.1371/journal.pone.0153975 (PMC4839727; doi:10.1371/journal.pone.0153975)

S1 File

Figure A. B16 relapse rates in Rag1$^{-/-}$ mice after ingenol mebutate treatment. Rag-1$^{-/-}$ mice (B6.129S7-Rag1^{tm1Mom}/J ; Jackson Laboratory, Bar Harbor, ME, USA) bred in-house at QIMR B and C57BL/6 mice (ARC, Perth, Australia) were injected s.c. with B16 cells (5 x 10^5 cells per mouse in 50 µl medium) on day -3. Relapse rates were not significantly different for ingenol mebutate treated Rag1$^{-/-}$ and C57BL/6 mice, log rank statistic, p=0.37.

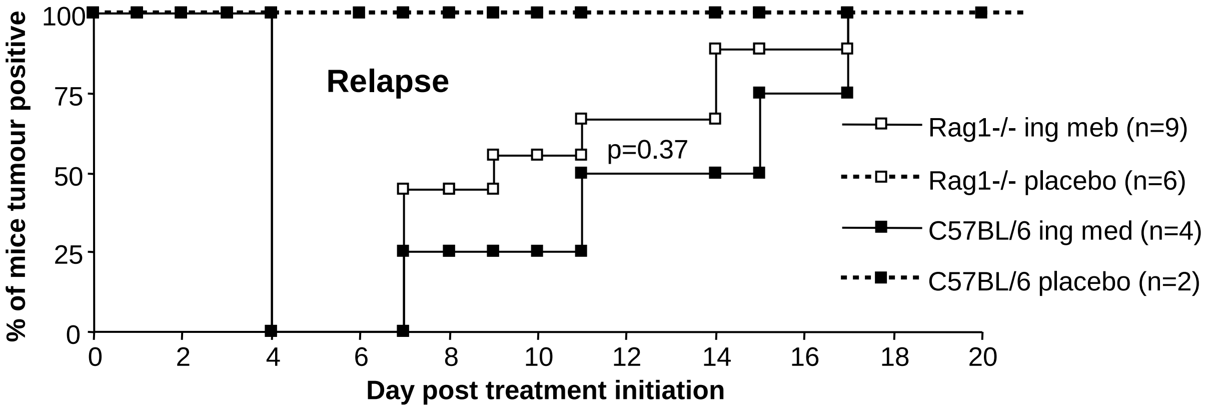

Figure B. To explore a role for ADCC in the B16 model [Challacombe, et al., 2006], relapse rates were examined in Fc receptor common gamma chain deficient mice (FcγR$^{-/-}$) mice (B6.129P2-Fcer1g^{tm1Rav} N12, purchased from Taconic Germantown, NY, USA). Relapse rates were slightly higher (but not significantly) (a, Relapse), and survival was slightly (and significantly) lower (b, Survival) in FcγR$^{-/-}$ mice. However, placebo treated B16 tumour grew lightly faster in FcγR$^{-/-}$ mice (c, Growth). As the slightly higher relapse rates could thus simply be due to more robust growth of B16 tumours in FcγR$^{-/-}$ mice, these experiments provided no conclusive insights into the role of ADCC in this model.

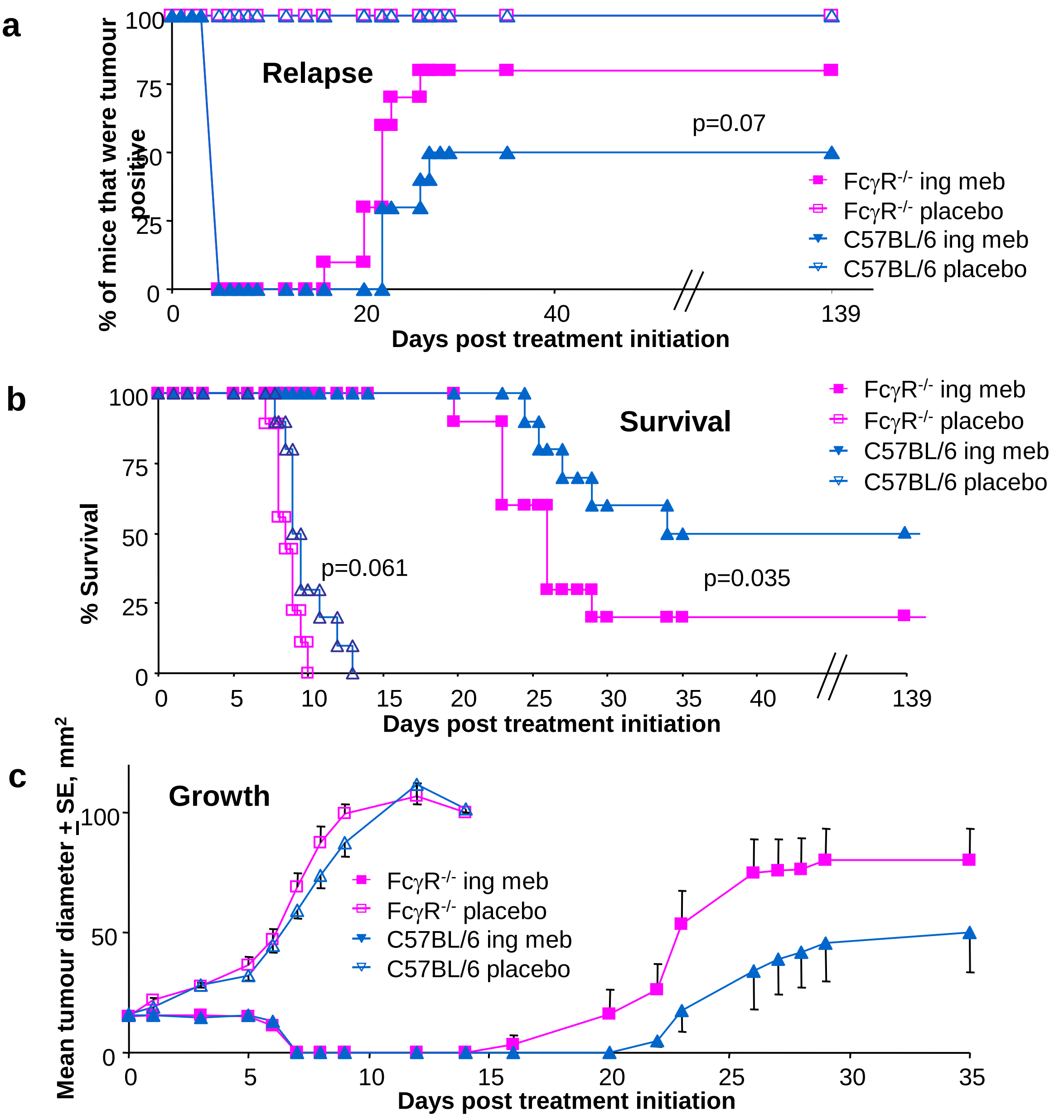

Figure B legend. B16 relapse , survival and growth in FcγR$^{-/-}$ mice after ingenol mebutate treatment. (a) Relapse rates of B16 tumours in FcγR$^{-/-}$ mice after ingenol mebutate (FcγR$^{-/-}$ ing meb) or placebo treatment (FcγR$^{-/-}$ placebo) and C57BL/6 mice after ingenol mebutate (C57BL/6 ing meb) or placebo treatment (C57BL/6 placebo) (n=9/10 mice per group). (b) Survival rates for the same mice described in a. A death event was recorded when the tumour reached 100 mm^2. Statistics by log rank. (c) Tumour growth curves for the same mice described in a. When a tumour reached ≈100 mm^2 the mouse was euthanized and for subsequent time points the value of 100 mm^2 was retained for that mouse in the calculation of the mean tumour diameters; thus n is the same for each time point.

Figure C. The role of anti-cancer antibodies was further investigated in B cell deficient µMT mice. No relapse events were seen after ingenol mebutate treatment of B16 tumours grown in µMT mice, whereas a 36% relapse rate was seen in C57BL/6 mice (a, Relapse). The reason for the lack of relapse in µMT mice was likely due to the very much slower growth rate of B16 tumours in µMT mice, when compared with C57BL/6 mice (b, Growth). The absence of antigen non-specific suppressor B cells may be responsible [Shah, et al.. Int J Cancer. 2005;117: 574-586] for this reduced rate of tumour growth µMT mice. The different growth rates in the absence of ingenol mebutate treatment, means µMT mice (like FcγR$^{-/-}$ mice) are of limited value for evaluating the role of antibodies in the anti-cancer efficacy of ingenol mebutate.

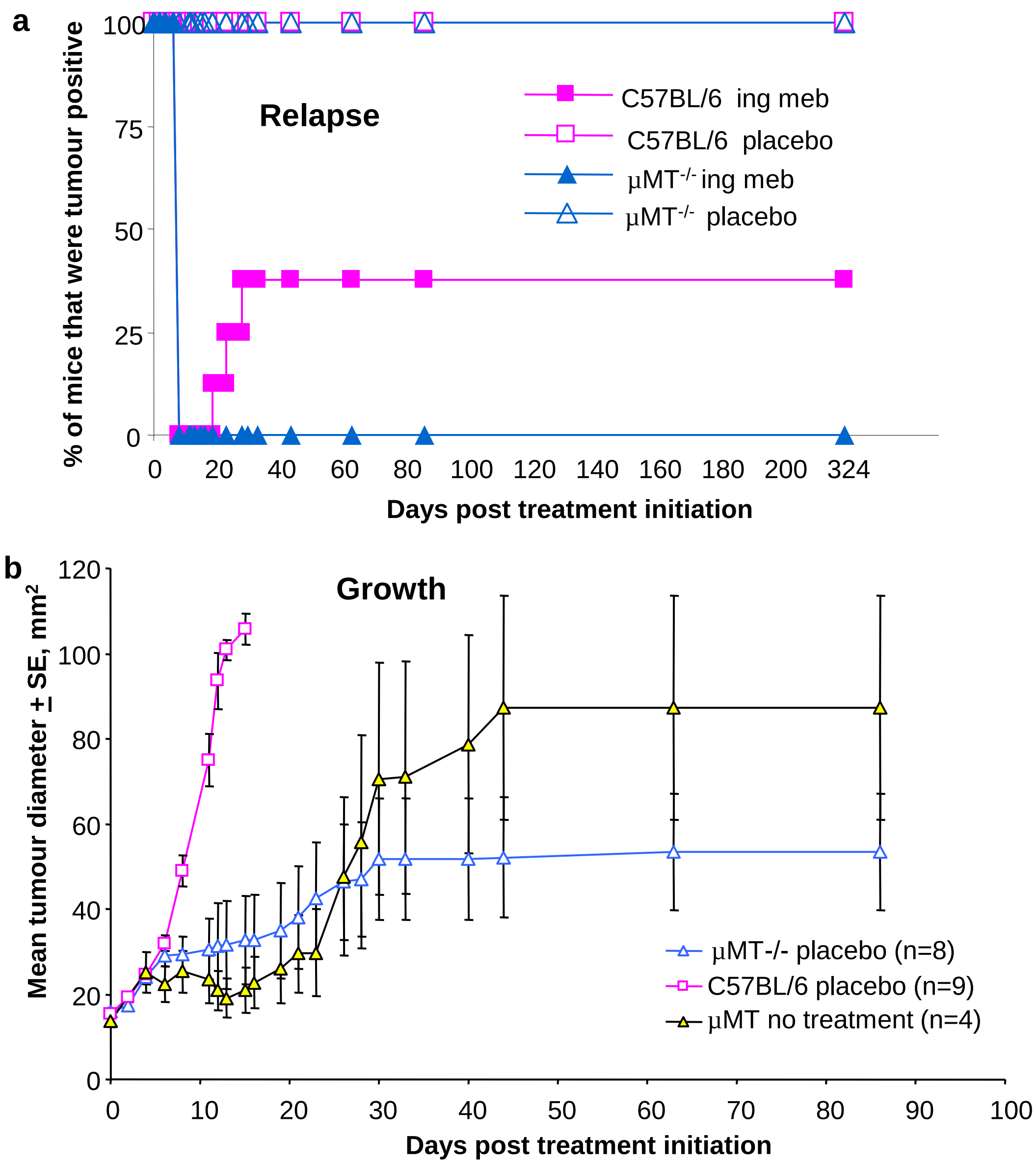

Figure C legend. B16 relapse rates in µMT$^{-/-}$ mice after ingenol mebutate treatment. (**a**) Relapse rates after ingenol mebutate and placebo treatment of B16 tumours in C57BL/6 mice and µMT$^{-/-}$ mice (n=8/9 mice per group). (**b**) Growth curves of B16 tumours in C57BL/6 and µMT$^{-/-}$ mice in the absence of ingenol mebutate treatment.

Figure D. Growth of B16 tumours in placebo treated MyD88$^{-/-}$ (n=7) and C57BL/6 mice (n=7). The first mice were euthanized on day 7. Tumour means for time points subsequent to day 7 were calculated using only those mice that remained alive.

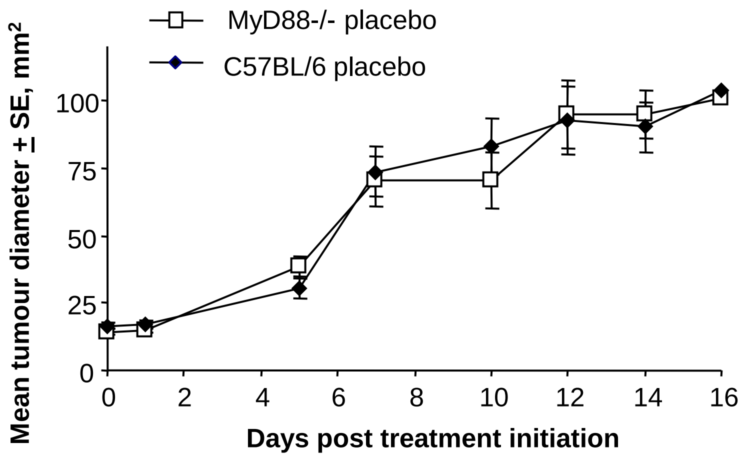

Figure E. Anakinra did not affect B16 growth. Growth data from mice shown in Fig. 2, illustrating that anakinra treatment does not affect growth of B16 tumours in C57BL/6 mice.

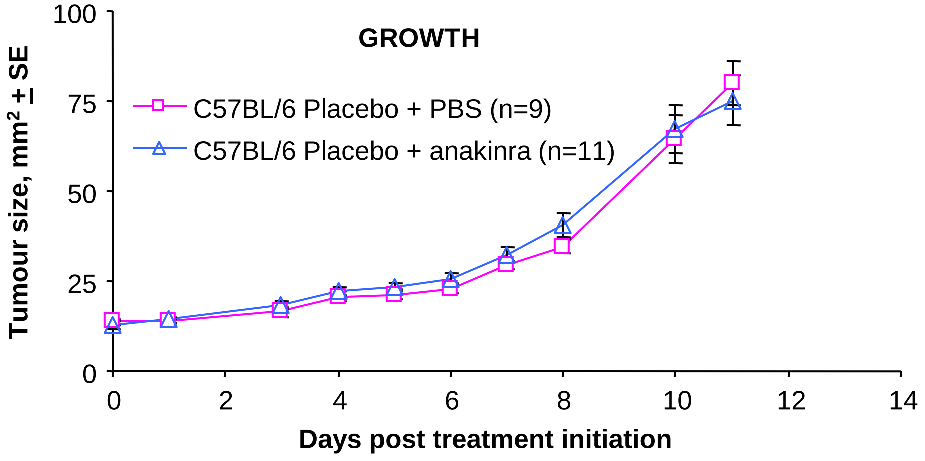

Figure F. High resolution images of ApoTag staining (with haematoxylin counterstain) of a neutrophil rich areas in the dermis of ingenol mebutate treatment sites 2 days post initiation of ingenol mebutate treatment. Green arrows show polymorphonuclear cells (predominantly neutrophils) and red arrows show cells staining with ApoTag.

Ingenol mebutate + PBS

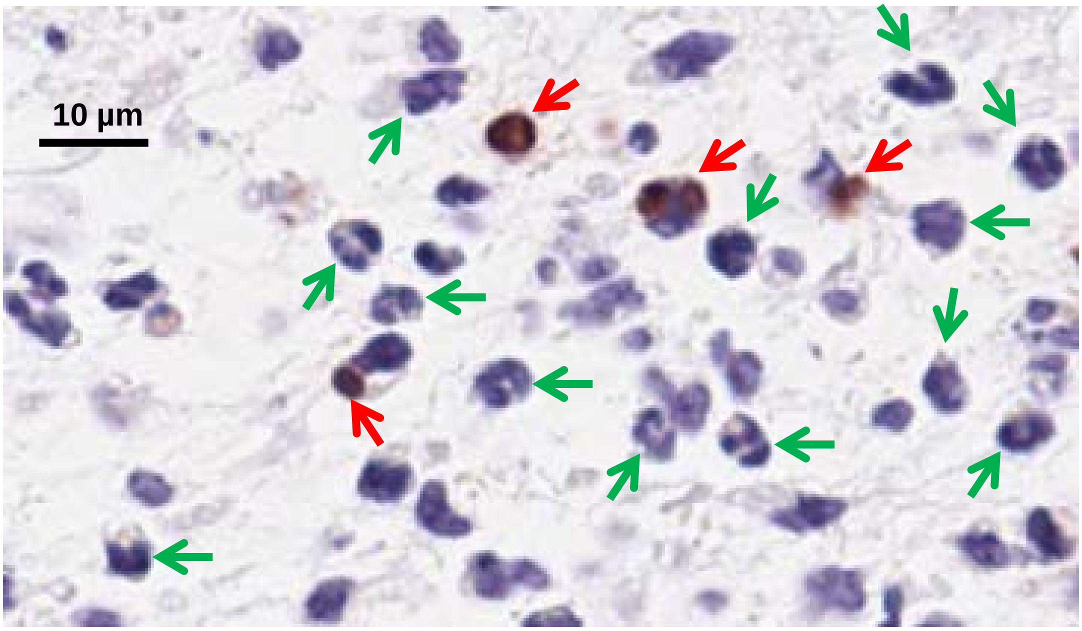

Ingenol mebutate + anakinra

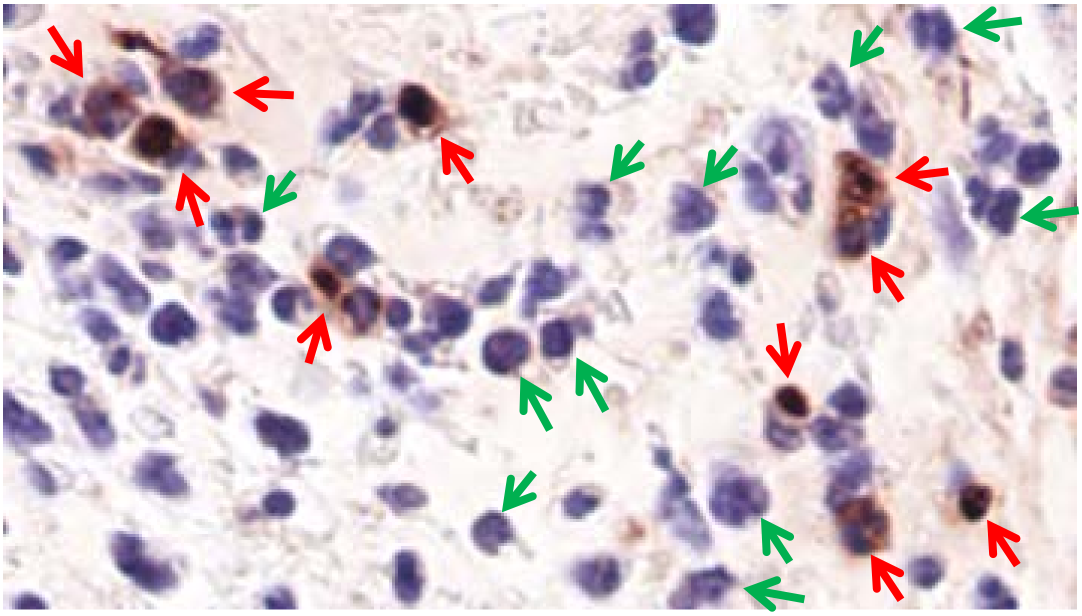

Figure G. IL-1α levels in skin and B16 tumours. Skin; naïve mouse skin (no B16 tumours) was excised from C57BL/6 and MyD88$^{-/-}$ mice and analysed for IL-1α levels (n=3 mice per group). B16 tumour; B16 tumours were excised from MyD88$^{-/-}$ and wild-type mice day 7 post inoculation, with extraneous tissue (eg skin) removed as much as possible and analysed for IL-1α levels (n=3 mice per group).

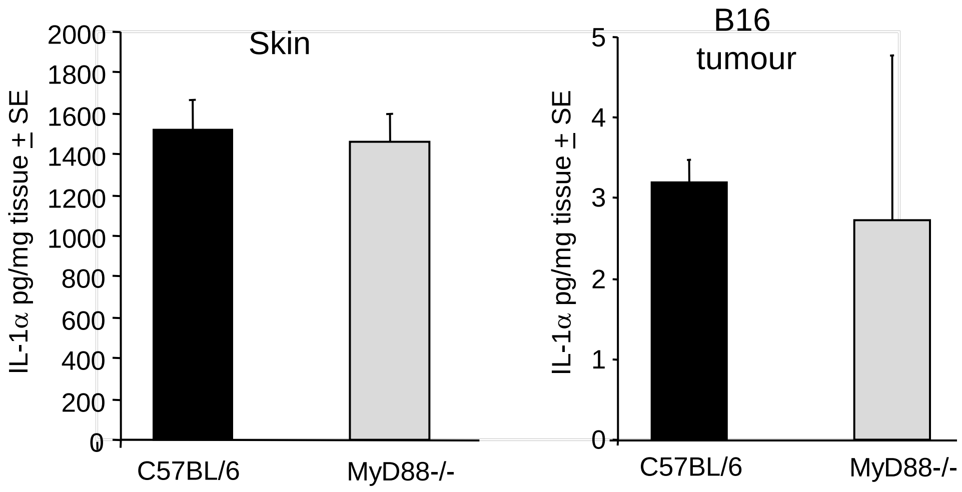

).

Figure H. Capase 1 in skin after inenol mebutate treatment. Mice (n=2 per time point) were treated once with ingenol mebutate on a shaved skin area (≈0.5 - 1 mm^2) on the back. At the indicated times the mice were euthanized and a scalpel blade was used to gently scrap the surface of the skin, with material placed into 450 µl 10 mM Tris pH7, with 0.1% Igepal and protease inhibitor cocktail at 4ºC. The preparation was sonicated (1 min x 5) and debris removed by centrifugation at 50,000 g for 40 mins at 4ºC. The supernatant was analysed by immunoblotting using anti-Caspase-1 p20 (Casper-1 clone, Adipogen), anti-Caspase-1 p10 (A-19 clone, Santa Cruz) and an anti-GAPDH (polyclonal mouse, BioScientific) loading control.

Ingenol mebutate treatment resulted in substantial increase in pro-caspase-1 protein levels, and the clear presence of the active caspase p20 and p10 species, illustrating caspase-1 activation [Guey et al.. Proc Natl Acad Sci U S A. 2014;111: 17254-17259].

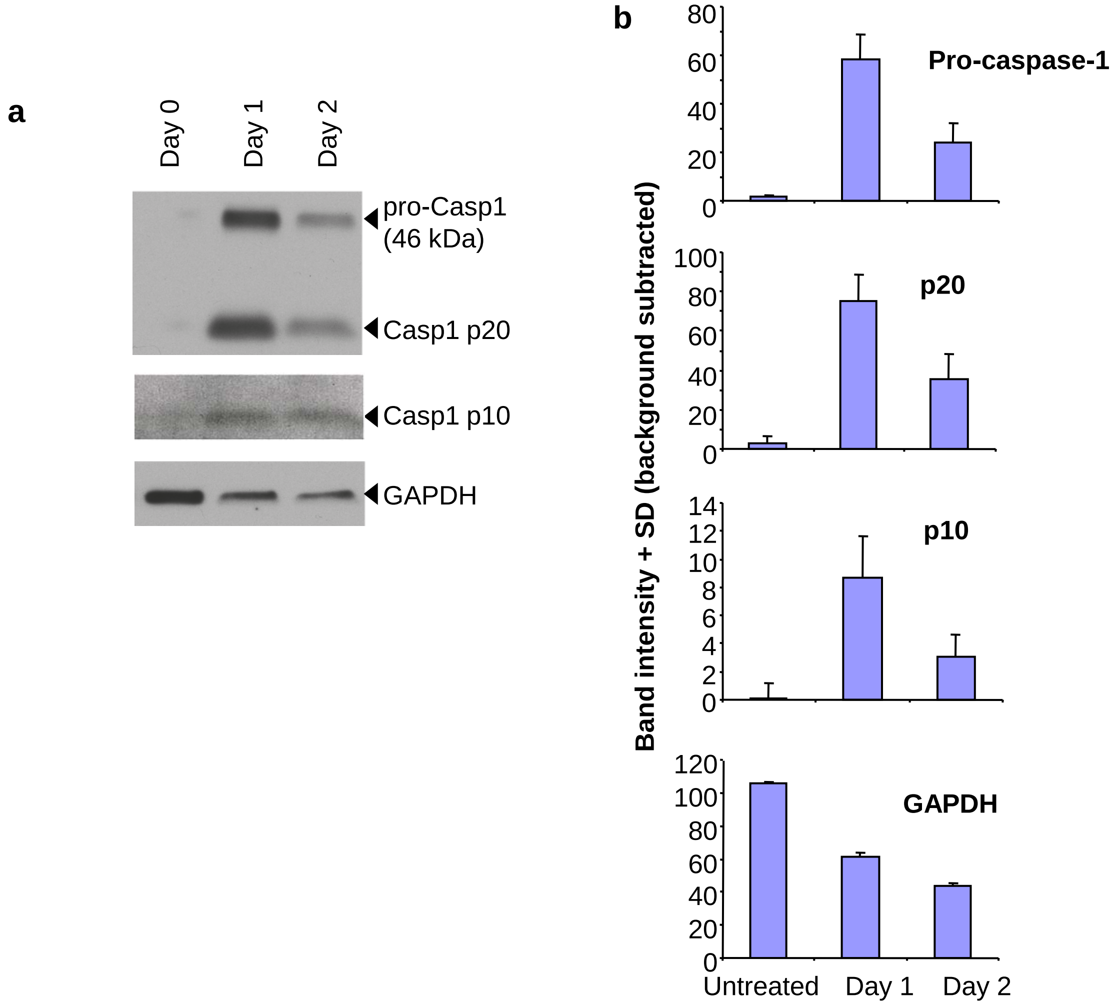

Figure J legend. (**a**) Western blot of skin samples at the indicated times after ingenol mebutate treatment (**b**). Densitometry of bands shown a. Mean of 2 exposures.

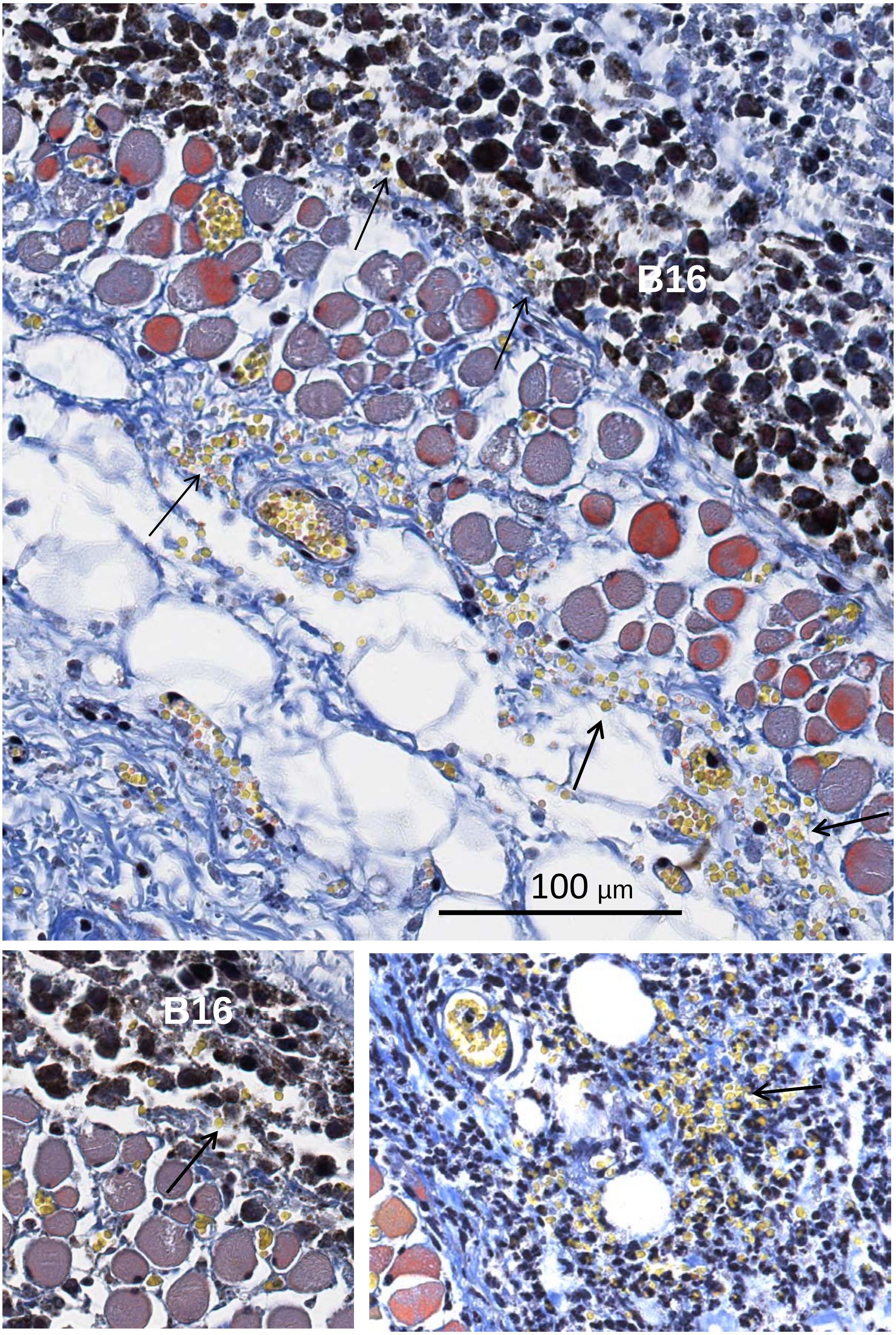

Figure I. Haemorrhage post ingenol mebutate treatment. Martius Scarlet Blue (MSB) staining of treatment sites 2 days post initiation of ingenol mebutate treatment. MSB is designed to stain fibrin (red), but also stains red blood cell yellow (arrows), thereby providing a clear demonstration of haemorrhage. The black B16 cells (melanin/melanosomes) are also clearly visible (B16). Neutrophils infiltrates are also evident (dark blue polymorphonuclear morphology, bottom right image).

Figure J. The effects of anti-B16 anti-serum on relapse rates of B16 tumours after ingenol mebutate treatment. Mice (n=5 per group) were treated with ingenol mebutate or placebo plus anti-B16 antiserum or medium. The antibody had an ELISA IgG end point titre of 1/36,000 (using B16 lysate as antigen) and was generated by immunising mice with B16 lysates formulated with Montanide ISA 720 (Le et al., 2009); 80 µl was adoptively transferred on day 0 by i.p. injection.

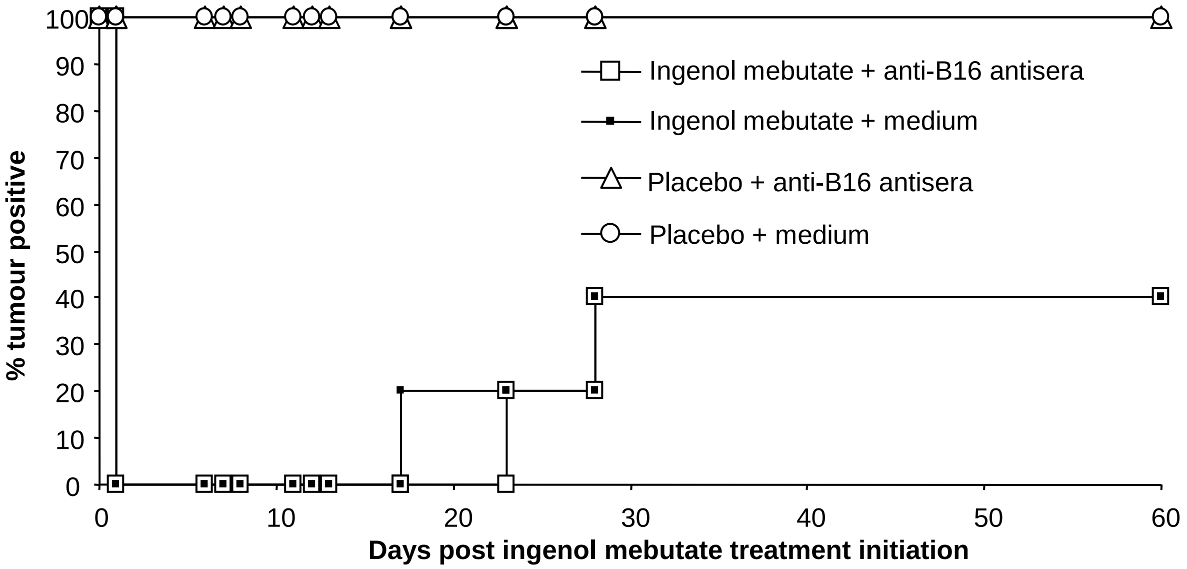

Supplement: S1 File — B16 relapse rates in Rag1-/- mice after ingenol mebutate treatment (Figure A). B16 relapse, survival and growth in FcγR-/- mice after ingenol mebutate (Figure B). B16 relapse rates in μMT-/- mice after ingenol mebutate treatment (Figure C). Growth of B16 tumours in placebo treated MyD88-/- and C57BL/6 mice (Figure D). Anakinra did not affect B16 growth (Figure E). High resolution images of ApoTag staining (Figure F). IL-1α levels in skin and B16 tumours (Figure G).) Capase 1 in skin after ingenol mebutate treatment (Figure H). Haemorrhage post ingenol mebutate treatment (Figure I). The effects of anti-B16 anti-serum on relapse rates of B16 tumours after ingenol mebutate treatment (Figure J). (PDF) [file pone.0153975.s001.pdf]
